# Cyanoacrylate vs. sutures in clean and clean-contaminated surgical wounds – a randomised control study

**DOI:** 10.1515/iss-2023-0060

**Published:** 2024-03-07

**Authors:** Kumkod Aravind, Chapparadallimath G. Sunil, Shruthi Chandrasekar, Shirol S. Shidlingappa, Vijay Kamat, Mukund Kulkarni, Sanganal P. Balachandra, Aswathy Chandran, Ramesh Vaidyanathan

**Affiliations:** Department of Surgical Gastroenterology Surgery, 29214 KIMS, Hubballi, Karnataka, India; Department of General Surgery, 37266 SDM College of Medical Sciences and Hospital, Dharwad, Karnataka, India; Department of Plastic Surgery, 28730 AIIMS, New Delhi, India; Department of Plastic Surgery, 29214 KIMS, Hubballi, Karnataka, India; Department of General Surgery, 29214 KIMS, Hubballi, Karnataka, India; Department of Plastic Surgery, 584304 Marsleeva Medicity Palai, Kottayam, India; Trauma Surgery and Critical Care, 28730 JPNATC AIIMS, New Delhi, India

**Keywords:** cyanoacrylate, sutures, adhesive glue, VAS score, ASEPSIS score, Modified Hollander Cosmesis score

## Abstract

**Objectives:**

Various techniques of closure of surgical incisions have been described ranging from various suture materials, staples and tapes to adhesive compounds. Cyanoacrylate is an adhesive compound available for surgical incision closure. Although sutures have been the preferred universal choice for surgical incision closure, glue is gaining popularity in specific places like pediatric injuries, facial injuries, laparoscopic incision closure, etc. This study aimed to compare the results between the application of cyanoacrylate and conventional suturing.

**Methods:**

In this randomized control study, patients were divided into two groups of 100 each. The surgical incisions were closed using cyanoacrylate glue in Group A patients and polyamide (EthilonTM 2-0) in Group B patients. Post-operative pain was assessed using Visual Analogue Scale on the first, third, and seventh day. The wounds were evaluated for complications on post-op days 1, 3, 7, and 30 using the ASEPSIS score. Cosmetic outcome was assessed at the end of first month using the Modified Hollander Cosmesis Scale.

**Results:**

Post-operative pain was significantly less in the glue group on days 1, 3, and 7. Wound infection with dehiscence occurred in 4 cases (4 %) in Group A and one patient (1 %) in Group B, which was statistically insignificant. There was no significant difference in cosmetic outcomes in either Group.

**Conclusions:**

Cyanoacrylate is a good alternative to sutures in skin closure of clean and clean-contaminated surgical wounds.

## Introduction

Wound healing and wound care have always fascinated surgeons. Advances in wound management have evolved from simple dressings to complex microvascular reconstructions. Management of surgically created incised wounds has advanced from simple sutures through staples to least invasive tissue adhesives. An ideal method of skin closure should be simple, easy, quick, cost-effective, painless, non-tissue reactive, non-allergic, reproducible with good results, leaves no marks of the material *per se,* and gives an aesthetic scar.

Various suture materials are commercially available for wound closure. These materials have evolved from naturally available synthetic materials like silk and catgut to synthetic materials like polypropylene and polyamide. Skin suturing is a skill-based procedure and requires anaesthesia. Suture removal is an additional procedure and may require anaesthesia or sedation, especially in children. Additional drawbacks include tissue reaction, suture line abscess or granuloma, and cross-hatch marks.

On the other hand, tissue adhesives are equally effective in skin closure without anaesthesia and additional suture removal procedures. They leave no cross-hatch marks. However, allergy to the molecule (various forms of cyanoacrylate) has been an issue along with wound infection due to the seepage of the glue into the raw surface or wound margin.

Although tissue glue has been studied for its uses in facial injuries, injuries in children, and laparoscopic incisions, it has not been routinely used in abdominal incisions and emergencies. Tissue glue (cyanoacrylate) is a recent and advanced method for closing surgical skin incisions. So, it is necessary to analyse the cosmetic outcome, wound strength, and infection rate compared to sutures to choose the most suitable method of skin closure for each case undergoing surgery.

Hence, we conducted a prospective randomised study to compare the efficacy of tissue adhesive (cyanoacrylate) in wound closure in clean and clean-contaminated surgeries done electively or as an emergency.

## Materials and methods

This randomised controlled study was conducted at a tertiary care hospital of an LMIC from December 2016 to June 2018. Institutional ethical clearance was taken. The objective was to compare *n*-butyl-2-cyanoacrylate vs. non-absorbable monofilament polyamide suture 2-0 closure in the surgical wound for postoperative pain, the incidence of local wound complications like wound dehiscence and wound infection rates and cosmetic outcome.

The study included all patients aged 12–70 years who underwent elective or emergency surgery for clean or clean-contaminated procedures. Informed written consent was obtained from all patients. Computer-generated block randomisation divided them into two groups. In Group A (n=100), the wound closure was done with cyanoacrylate, whereas in Group B (n=100), the closure was achieved with interrupted polyamide sutures.

Patients with a known past/family history of keloid formation, hypertrophic scars, or any history of allergy to cyanoacrylate (if available) were excluded from the study. Traumatic and septic wounds were also excluded from the study.

After surgery and confirmed hemostasis, subcutaneous closure was achieved using 2-0 Vicryl in both groups to relieve tension, close dead space, and oppose wound edges. Skin edges were approximated & closed using *n*-butyl-2-cyanoacrylate (ENDOCRYLTM/TRUSEALTM) in Group A (adhesive group) and with 2-0 polyamide mattress sutures in Group B. Cyanoacrylate was applied in a single layer keeping the wound edges well approximated with forceps.

Postoperative pain was assessed at 24 h, 72 h, and postoperative day 7 using the Visual Analogue Score [1]. Wound outcome (discharge/dehiscence/infection) was evaluated for complications at postoperative days 1, 3, 7, and then at 1-month intervals using ASEPSIS score [2].

It is an objective method to evaluate post-operative wound and is a given a score from 0 to 10 according to proportion of wound involved and presence of (i) serous exudate (ii) erythema (iii) purulent exudate and (iv) any separation of deep tissues. Additionally, points are given for treatment given as illustrated in the figure below.

**Figure:**
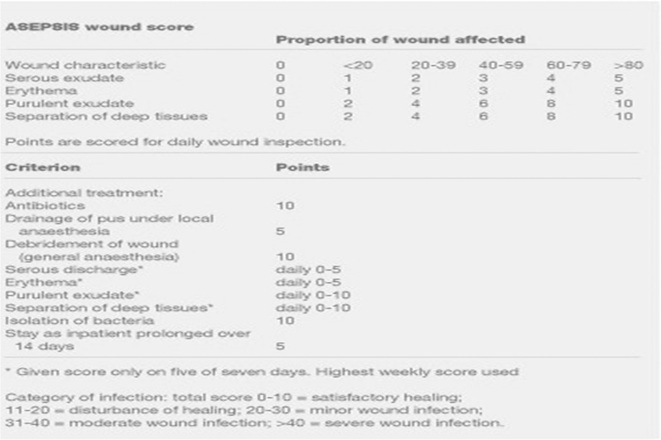


The cosmetic appearance of the wound was assessed after one month using the Modified Hollander Cosmesis Scale [3]. In brief, wound will be assigned 0 or 1 point each for the presence or absence of the following:

1. Step off the borders, (0 for yes, 1 for no)
2. Contour irregularities – puckering, (0 for yes, 1 for no)
3. Wound margin separation, (0 for yes, 1 for no)
4. Wound edge inversion, (0 for yes, 1 for no)
5. Excessive wound distortion, (0 for yes, 1 for no)
6. Good overall appearance (0 for poor, 1 for acceptable)

Wound with a score of 6 are considered to have an optimal cosmetic appearance, other’s suboptimal appearance.

**Figure:**
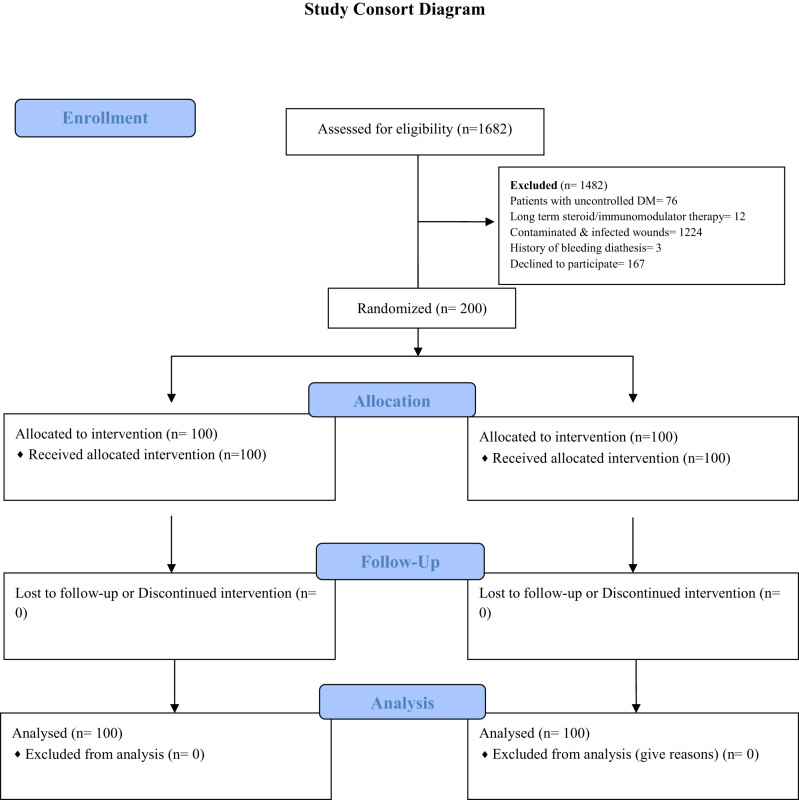


## Results

Out of the 200 patients in the study, 140 were males and 60 were females. The most common operative procedure in this study was Lichtenstein tension-free meshplasty for inguinal hernia repair (n=60, 30 %), followed by open appendectomy (n=52, 26 %), laparoscopic cholecystectomy (30) and other hernia repairs (19). Table 1 shows the distribution of various surgical procedures between the two groups.

**Table 1:** Procedures done for the participants.

Procedures	Group A	Group B
	(n=100)	(n=100)
Lichtenstein’s tension free meshplasty for inguinal hernia	28	32
Meshplasty for other ventral hernia	1	11
Open appendicectomy	16	36
Laparoscopic cholecystectomy	28	4
Laparoscopic TEP repair	18	4
Neck surgeries	2	4
Other laparoscopic procedures	3	1
Other hernia repair (TAPP repair, anatomical repair)	2	4
Others	2	4

TEP, total extraperitoneal repair; TAPP, transabdominal preperitoneal repair.

**Figure 1:**
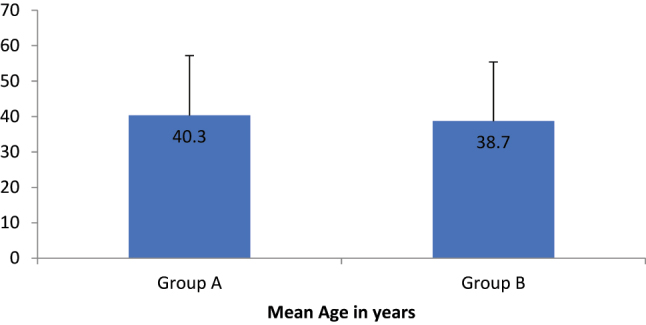
Mean age of study participants (n=200).

**Figure 2:**
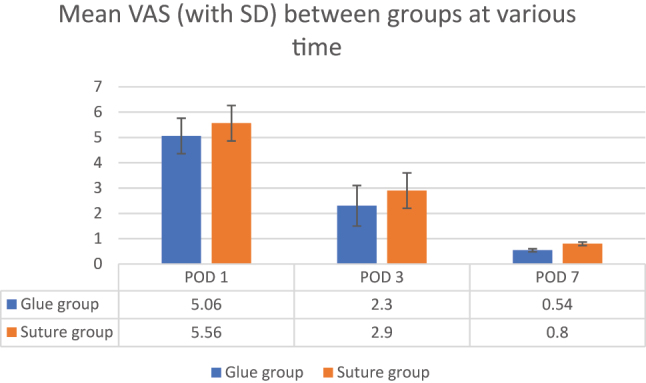
Mean VAS [Visual Analogue Score] with standard deviation of study participants at various time point between groups (n=200).

The mean age in the adhesive group was 40.3 years while in the suture group, it was 38.7 years (Figure 1). The difference however was not significant (p value=0.51) (Figure 1). The postoperative pain was assessed using the Visual Analog Scale (VAS). Pain was significantly less in the adhesive group on postoperative days 1, 3, and 7 (Figure 2).

ASEPSIS score was used to assess local wound complications on the first, third, seventh, and 30th postoperative days (Table 2). There was no significant difference between the two groups concerning local complications as shown in Table 3.

**Table 2:** Assessment of pain score using VAS between groups at various times.

Pain assessment using VAS	Group A (n=100)	Group B (n=100)	p-Value
	Mean (SD)	Mean (SD)	
POD 1	5.06 (0.7)	5.56 (0.7)	0.001
POD 3	2.3 (0.8)	2.9 (0.7)	0.001
POD 7	0.54 (0.06)	0.80 (0.07)	0.01

POD, post-operative day; VAS, Visual Analogue Score; SD, standard deviation.

In the adhesive Group (A), four patients (4 %) developed complications while only one patient in Group B developed complications in the form of minor wound infection (Tables 3 and 4).

**Table 3:** Comparison of wound characteristic using ASEPSIS score of either group at various study points.

Total ASEPSIS score	Total patients in Group A	Total patients in Group B	p-Value
**Day 1**			

0	99	100	0.31
10	1	0	

**Day 3**			

0	98	100	0.39
8	1	0	
10	1	0	
20	0	1	

**Day 7**			

0	97	100	0.22
30	2	0	
35	1	0	

**1 Month**			

0	100	100	**–**

p value is based on Chi-square test.

**Table 4:** Details of complications.

Complications	Number of patients in Group A	Number of patients in Group B	p-Value
Wound dehiscence	1	0	0.28
Wound dehiscence with minor infection	2	0	
Wound dehiscence with moderate infection	1	0	
Minor wound infection	0	1	

p value is based on Chi-square test.

**Figure 3:**
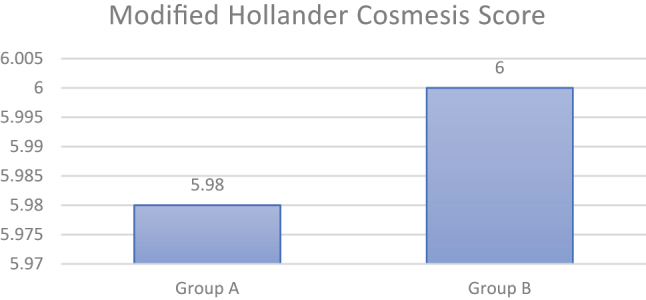
Mean cosmetic outcome between groups at the end of one month.

There was no significant difference found between cosmetic outcomes among either group at the end of 1 month (p-value 0.15) (Figure 3 and Table 6).

## Discussion

Tissue glue has been extensively studied for diverse indications, including tissue adhesion, wound closure, hemostasis, closure of CSF leaks, vascular embolization, and skin graft application. Although cyanoacrylates have many external uses, many studies report their use in various internal situations, such as the repair of broncho-pleural fistulae [4], mesh fixation for an inguinal hernia [5], myocardial tears, and to control bleeding from gastric varices by sclerotherapy using the tissue adhesive agent butyl cyanoacrylate [6].

**Table 5:** Details of interventions for the complications.

Complications	Number of patients in Group A	Number of patients in Group B	p-Value
Drainage of pus with regular cleaning and dressing and oral antibiotics	2	0	0.28
Drainage of pus with regular cleaning and dressing and oral antibiotics and secondary suturing done on day-14	1	0	
Reapplication of glue on day-1 and delayed primary suturing on day 3	1	0	
Regular cleaning and dressing with antibiotics	0	1	

p value is based on Chi-square test.

**Table 6:** Modified Hollander Cosmesis score of study participants at end of 1 month (n=200).

Modified Hollander Cosmesis score	Total patient in Group A	Total patients in Group B	p-Value
5	2	0	0.15
6	98	100	

p value is based on Chi-square test.

When the cyanoacrylate monomer comes into contact with skin moisture, it chemically changes into a polymer that binds to the top epithelial layer. This polymer forms a cyanoacrylate bridge, binding the two wound edges together and allowing normal healing below [7]. Short-chain derivatives have more tissue toxicity than long-chain derivatives [8]. Higher cyanoacrylates like *n*-butyl-2-cyanoacrylate and isoamyl 2-cyanoacrylate degrade slower than those with shorter side-chained ones. These materials are less histotoxic due to their slow degradation [9]. As they break down slowly, applying multiple continuous layers between two tissue surfaces is not advisable. The adhesive is applied to the junction after proper approximation of tissue edges. When the edges are improperly approximated, the adhesive material may enter the wound, interfering with edge approximation and leading to wound dehiscence (Figure 4a–c).

**Figure 4:**
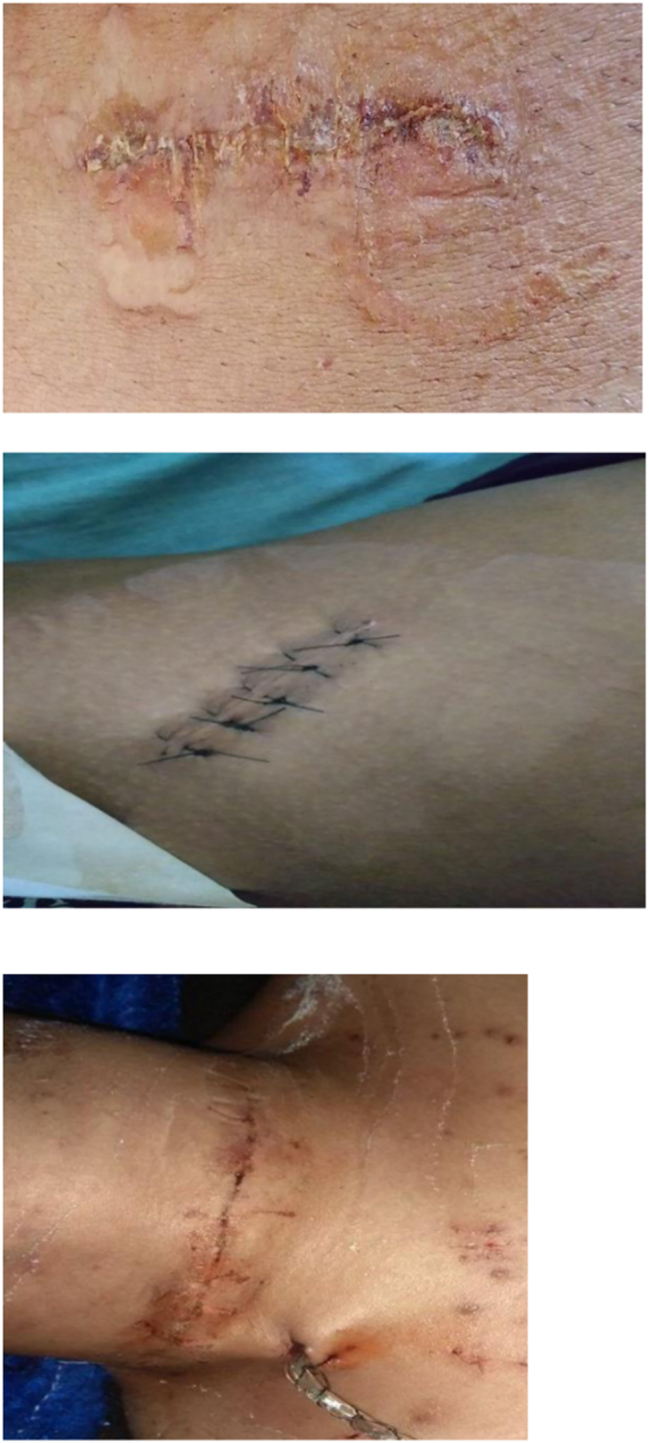
Clinical photographs: immediate post-operative picture.

**Figure 4b:**
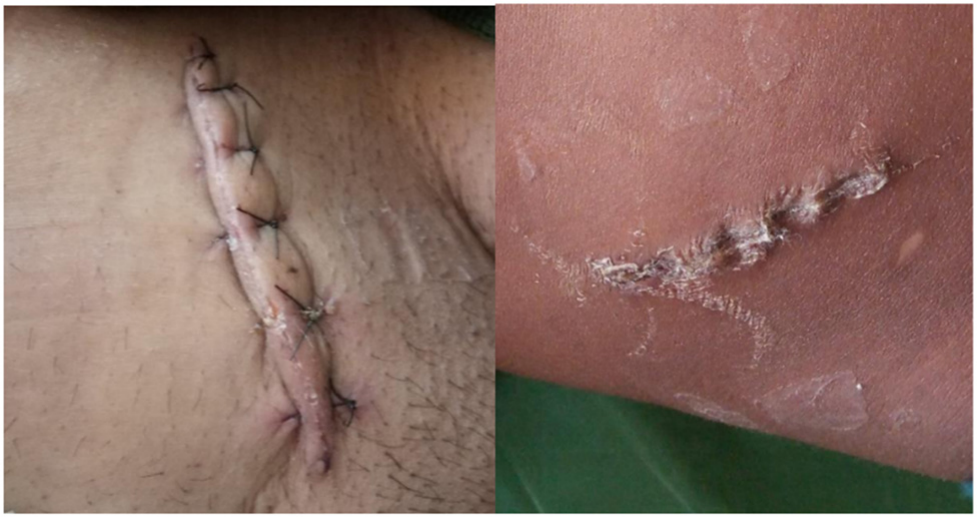
Clinical photographs: post-operative day 3 picture.

**Figure 4c:**
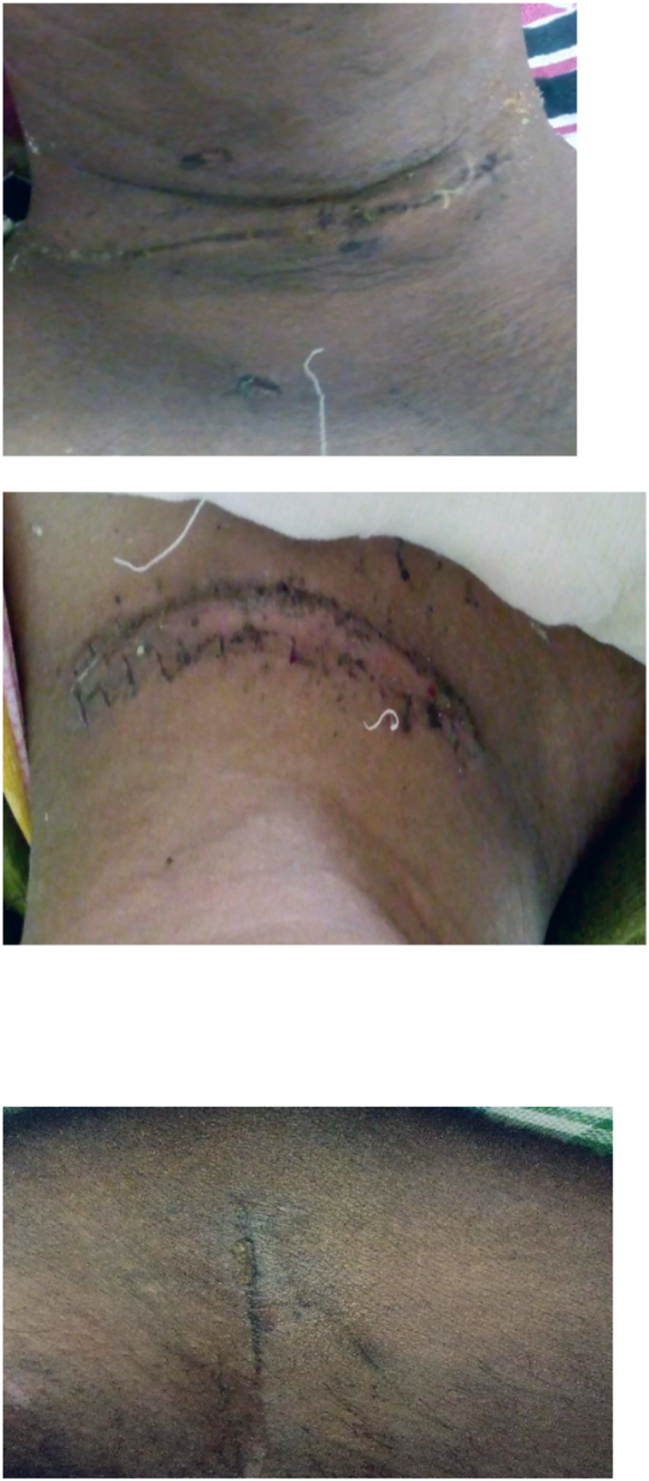
Clinical photographs: post-operative day 7 picture.

**Figure 4d:**
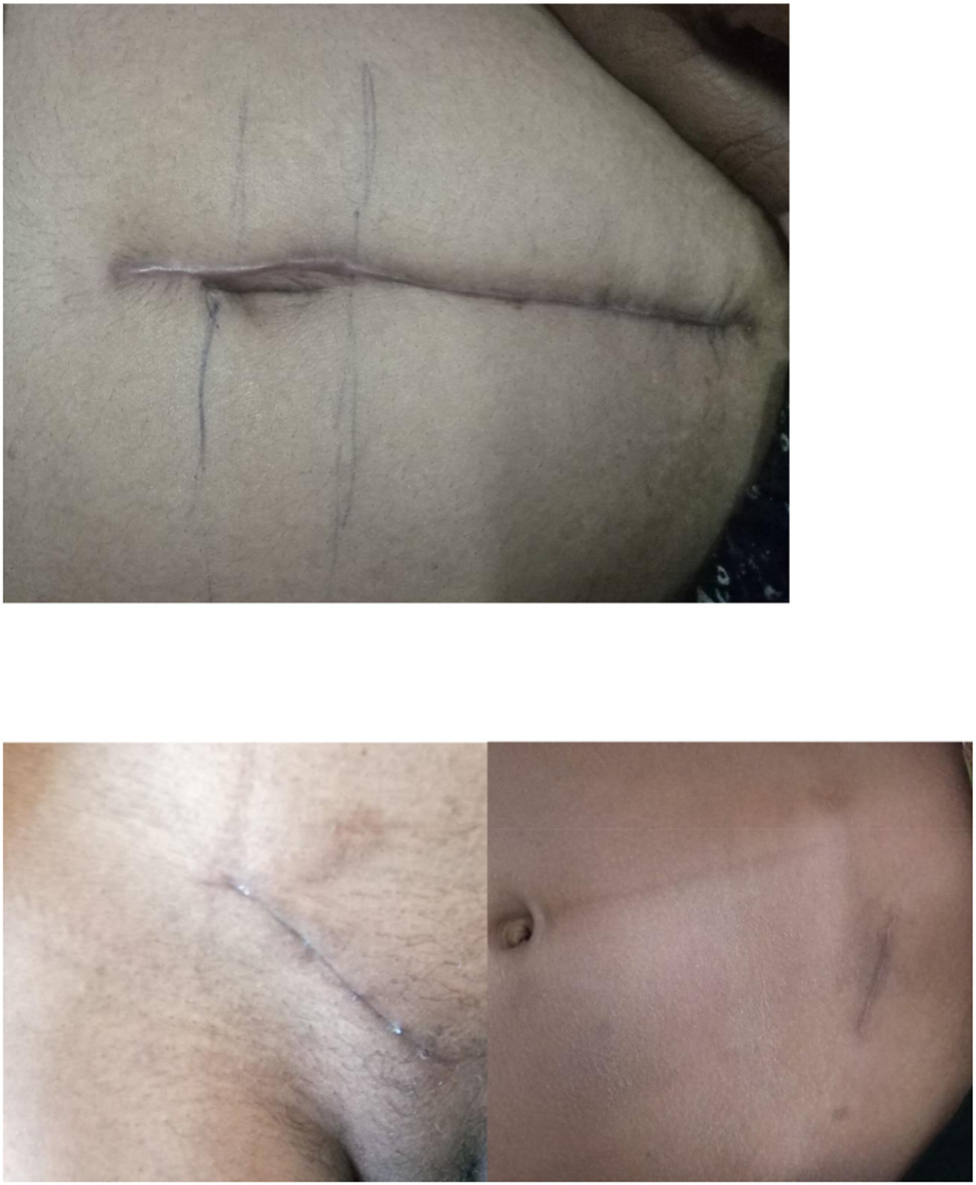
Clinical photographs: wound at the end of 1 month.

**Figure 4e:**
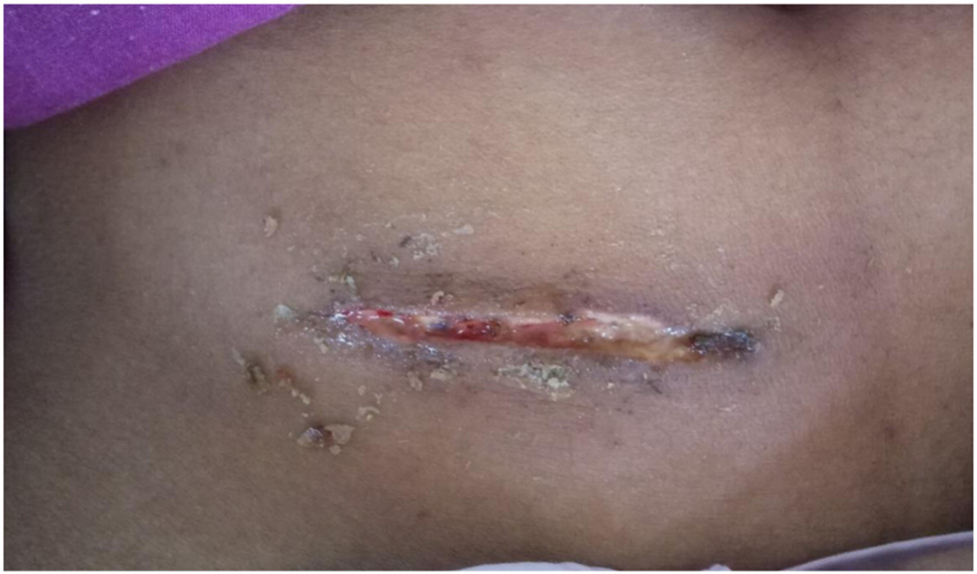
Clinical photographs: wound complication in glue group.

There are varying reports regarding cyanoacrylate glue’s antibacterial properties [10]. The glue has a bacteriostatic effect against Gram-positive bacteria, while no activity has been reported against Gram-negative bacteria. Since its first use in 1989, topical tissue adhesives have become a popular method for closing skin lesions, laparoscopic incisions and trauma-induced lacerations, and thyroidectomy incisions, i.e. in areas of low tension [11]. None of the available studies have evaluated glue use in laparotomy wounds and emergency surgeries. So, this study was conducted to evaluate the suitability of glue for these cases as well.

The advantages of using cyanoacrylate are that it acts as a waterproof dressing, prevents cross-hatch marks, and helps reduce follow-up visits, as there is no need for suture removal. As they do not require needles, accidental needlestick injuries are also prevented. However, cyanoacrylates have certain disadvantages, such as low tensile strength, inability to control bleeding, chances of adhesive seepage if edges are not adequately approximated, and the possibility of tissue reaction.

In our study, post-operative pain in the adhesive (glue) group was significantly lower than in the suture group at 24 h, 72 h, and on day 7, favoring the glue. Previous studies have compared the post-operative pain using Visual Analogue Scales of 0–100 and demonstrated less pain in the adhesive glue group [12], [13], [14].

4 % (4 cases) of the patients in the adhesive glue group developed wound infection with dehiscence (Figure 4e). After a detailed analysis and re-evaluation, we found that two of the four dehiscence with infection in the adhesive group were due to faulty technique, i.e. seepage of the adhesive into the wound margin. Two underwent drainage of pus with regular cleaning and dressing along with oral antibiotics, where the wound healed with secondary intention. One patient underwent drainage of pus with regular cleaning and dressing along with oral antibiotics, and the wound was closed by secondary suturing on day 14. In the last one, glue was reapplied on day one but dehisced again, and delayed primary suturing was done on day 3 (Table 5).

Only one patient in suture group (B) developed a complication in the form of a minor wound infection which was managed with regular cleaning and dressing, and antibiotics. There was no significant difference between the two groups in terms of ASEPSIS score on day 1 (p=0.31), 3 (p=0.39), 7 (p=0.22), and on day 30 (Figure 4d). Qureshi et al. [15] reported two out of 102 cases (<2 %) of partial dehiscence after general abdominal and laparoscopic incisions. In a recent study by Chawada et al. [16], wound infection or dehiscence incidence was significantly lower in the adhesive group. This again highlights that although skin closure using glue is quick and easy, proper technique must be followed to prevent complications associated with its use.

Although our study did not reveal any significant difference in cosmetic outcome at one month using MHC score, Krishnamoorthy et al. [17] reported superior cosmetic outcomes at the end of the first and sixth week, whereas reports by others [18, 19] did not show significant cosmetic differences.

The scar matures over time and becomes better cosmetically as the scar becomes more mature. A long-term follow-up of the scar at six months, one year, and the end of 2 years is desirable to assess the cosmetic outcome of the scar as well as to analyze for scar hypertrophy, keloid formation, scar hyper/hypopigmentation and also manage these complications with appropriate intervention.

The study’s major limitation is the lack of long-term follow-up and assessment of the scar at the end of one month when the scar tends to be red and immature. Long-term follow-up of these patients for at least two years would be appropriate to study the actual cosmetic outcome difference among these groups.

## Conclusions

N-butyl cyanoacrylate provides an effective, reliable, safe, and cosmetically acceptable alternative to conventional sutures for surgical wound closure in clean and clean-contaminated surgery. Cyanoacrylate glue is less painful than conventional sutures with comparable local complication rates and cosmetic outcomes.
